# Oleic acid induces smooth muscle foam cell formation and enhances atherosclerotic lesion development via CD36

**DOI:** 10.1186/1476-511X-10-53

**Published:** 2011-04-12

**Authors:** Shuangtao Ma, Dachun Yang, De Li, Bing Tang, Yongjian Yang

**Affiliations:** 1 Department of Cardiology, General Hospital of PLA Chengdu Military Area Command, Chengdu 610083, PR China

## Abstract

**Background:**

Elevated plasma free fatty acid (FFA) levels have been linked to the development of atherosclerosis. However, how FFA causes atherosclerosis has not been determined. Because fatty acid translocase (FAT/CD36) is responsible for the uptake of FFA, we hypothesized that the atherogenic effects of FFA may be mediated via CD36.

**Results:**

We tested this hypothesis using cultured rat aortic smooth muscle cells (SMCs) treated with oleic acid (OA). We found that OA induces lipid accumulation in SMCs in a dose dependent manner. Rat aortic SMCs treated for 48 hours with OA (250 μmol/L) became foam cells based on morphological (Oil Red O staining) and biochemical (5 times increase in cellular triglyceride) criteria. Moreover, specific inhibition of CD36 by sulfo-N-succinimidyl oleate significantly attenuated OA induced lipid accumulation and foam cell formation. To confirm these results *in vivo*, we used ApoE-deficient mice fed with normal chow (NC), OA diet, NC plus lipolysis inhibitor acipimox or OA plus acipimox. OA-fed mice showed increased plasma FFA levels and enhanced atherosclerotic lesions in the aortic sinus compared to the NC group (both *p *< 0.01). This effect was partially reversed by acipimox (lesion area: OA: 3.09 ± 0.10 ×10^5 ^μm^2 ^vs. OA plus acipimox: 2.60 ± 0.10 ×10^5 ^μm^2^, *p *< 0.05; FFA: OA: 0.91 ± 0.03 mmol/L vs. OA plus acipimox: 0.78 ± 0.03 mmol/L, *p *< 0.05).

**Conclusions:**

These findings suggest that OA induces smooth muscle foam cell formation and enhances atherosclerotic lesions in part though CD36. Furthermore, these findings provide a novel model for the investigation of atherosclerosis.

## Background

Foam cell formation is an important process in the development of atherosclerosis [1]. Foam cells have been demonstrated to be of either macrophage or smooth muscle cell (SMC) origin [2]. Circulating cholesterol has been traditionally recognized as an important risk factor of atherosclerosis. Macrophages can easily be induced to transform into foam cells by loading modified low density lipoprotein (LDL) cholesterol donors, including ox-LDL. However, the method that induces SMC to form foam cell through cholesterol loading has been challenged [3]. Attempts to extensively load vascular SMC with cholesterol have been largely unsuccessful [4-6]. This phenomenon suggests that there is another mechanism underlying the formation of SMC-derived foam cells. To date, the mechanism by which vascular SMC could accumulate large stores of lipids remains elusive.

Cholesterol and triglyceride are the major components of atherosclerotic plaques. Cholesterol esters and triglycerides all contain fatty acids. Pilz *et al *[7] have reported that free fatty acids (FFA) are independently associated with cardiovascular mortality in subjects with coronary artery disease, indicating that FFA might promote the development of atherosclerosis. Although FFA is linked to the pathogenesis of atherosclerosis [8], the mechanism by which FFA regulates the development of atherosclerosis is still unknown [9].

Several studies have demonstrated that FFA might cause a significant amount of lipid accumulated in both hepatic [10] and skeletal muscle cells [11]. Fatty acid translocase (FAT/CD36), originally identified as glycoprotein IV on platelets, is an 88-kDa integral membrane protein that has multiple legends and is widely expressed in different cell types [12], including vascular SMCs [13]. Recent studies have shown that CD36 is also a crucial transporter for FFA [14]. Previous observations have suggested that CD36 deficiency may provide a more atherogenic environment to accelerate the development of atherosclerosis [13], causing a significant increase in fasting levels of FFA.

Therefore, we hypothesized that FFA might induce smooth muscle foam cell formation and enhance atherosclerotic lesion development via CD36. In the present study, we present a model of smooth muscle foam cell formation. We propose that elevated levels of FFA could increase the uptake of fatty acids in vascular SMC via CD36 and subsequently induce the formation of smooth muscle foam cells and accelerate the development of atherosclerotic lesions in ApoE-deficient (ApoE^-/-^) mice.

## Results

### Induction of vascular smooth muscle foam cell formation by oleic acid

**Figure 1:**
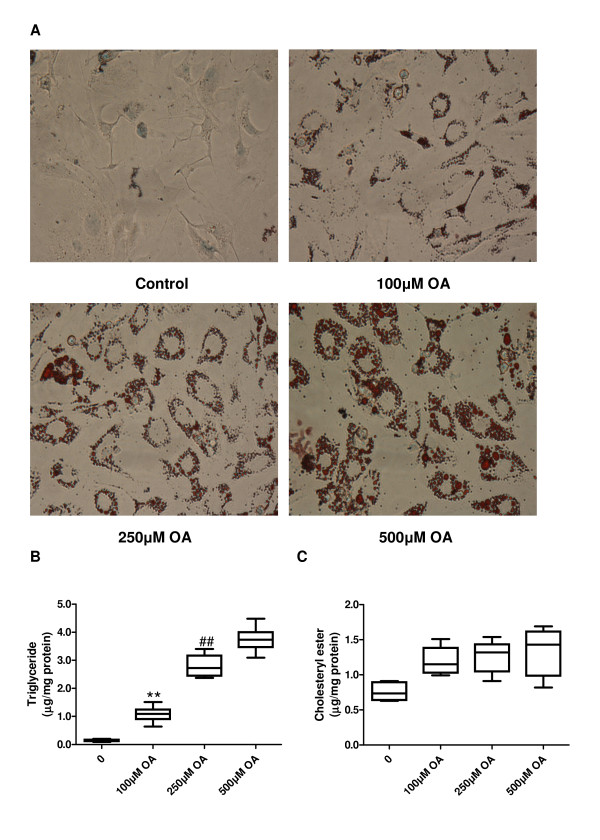
**Vascular smooth muscle foam cell formation is induced by OA treatment**. (A) Cellular lipid accumulation in rat aortic SMCs after incubation for 48 hours with indicated concentrations of OA. Magnification ×400. Triglyceride (B) and cholesterol ester (C) levels normalized to cellular protein content in rat aortic SMCs. Data are expressed as mean ± SEM. ** *p *< 0.01 compared with the control group, ^## ^*p *< 0.01 compared with the cells treated with 100 μmol/L of OA. OA, oleic acid; SMCs, smooth muscle cells.

We examined lipid accumulation by using Oil Red O staining (Figure 1A). After incubation with oleic acid (OA) for 48 hours, rat SMC assumed the morphological appearance of foam cells with significant lipid accumulation; in contrast, no lipid droplets were found in the untreated cells. The visual abundance of lipid droplets was grossly correlated with OA concentration in the treatment. Measurement of triglyceride and cholesterol esters confirmed cellular accumulation of lipids. Incubation with OA increased intracellular triglyceride levels in a dose-dependent manner (*p *< 0.01, Figure 1B). However, intracellular cholesterol esters were slightly, but not significantly, increased by incubation with OA at a concentration of 100 μmol/L and were not further increased by OA at higher concentrations (Figure 1C).

### OA-induced vascular smooth muscle foam cell formation is CD36-dependent

**Figure 2:**
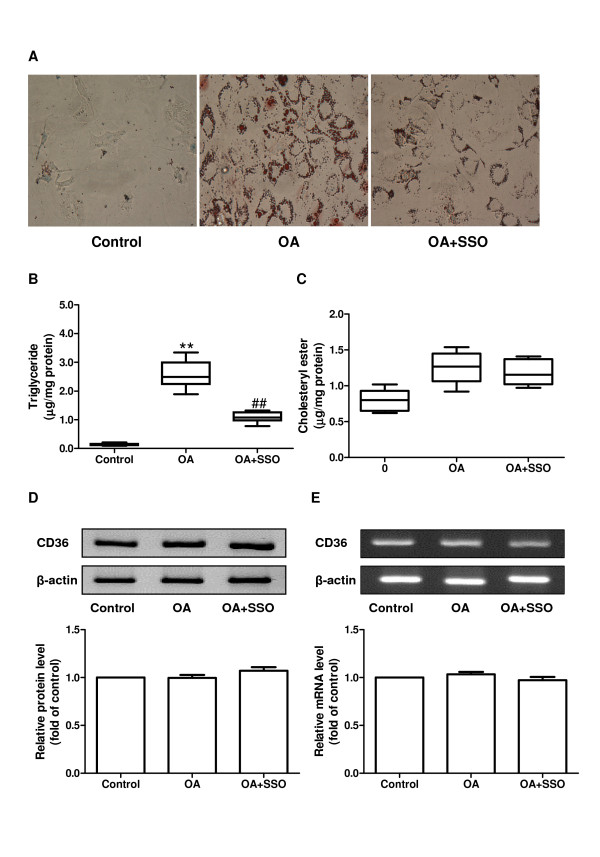
**OA-induced foam cell formation is CD36-dependent**. (A) Cellular lipid accumulation in rat aortic SMCs after incubation for 48 hours with vehicle (control), OA (250 μmol/L), or OA (250 μmol/L) plus SSO (250 μmol/L). Magnification ×400. Triglyceride (B) and cholesterol ester (C) levels normalized to cellular protein content in rat aortic SMCs. (D) CD36 was determined by western blotting using a specific antibody. Equal protein loading was confirmed using β-actin antibody. The graph below the blot shows quantification. (E) CD36 was determined by RT-PCR. Equal RNA loading was confirmed using β-actin primers. The graph below the blot shows quantification. Data are expressed as mean ± SEM. ** *p *< 0.01 compared with the control group, ^## ^*p *< 0.01 compared with the cells treated with OA alone. OA, oleic acid; SMCs, smooth muscle cells; SSO, sulfosuccinimidyl oleate.

When rat SMCs were incubated with OA (250 μmol/L) plus CD36 inhibitor sulfo-N-succinimidyl oleate (SSO), cellular lipid inclusions were significantly decreased compared with cells treated only with OA (Figure 2A). Biochemical measurements were consistent with the Oil Red O staining results; compared with the OA-treated SMCs, intracellular triglyceride levels were significantly reduced in cells treated with OA plus SSD (*p *< 0.01, Figure 2B). However, SSD did not affect intracellular cholesterol ester levels in cells treated with OA (Figure 2C). Additionally, intracellular triglyceride and cholesterol ester content remained constant in the SMCs treated with SSO only (data not shown). These results suggested that OA-induced vascular smooth muscle foam cell formation is CD36-dependent. Furthermore, the protein level of CD36 in vascular SMCs was not affected by either OA or SSO (Figure 2D). Similarly, neither OA nor SSO influenced the mRNA level of CD36 in vascular SMCs (Figure 2E).

### OA modulates vascular SMC phenotype

**Figure 3:**
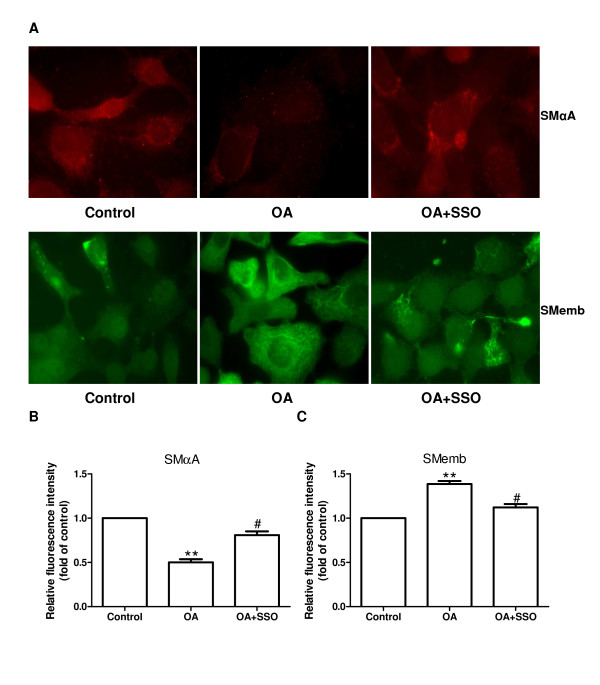
**OA modulates vascular SMC phenotype**. (A) Immunofluorescence staining of SMαA and SMemb proteins in rat aortic SMCs after incubation for 48 hours with vehicle (control), OA (250 μmol/L), or OA (250 μmol/L) plus SSO (250 μmol/L). Magnification ×400. (B, C) Summarized data showing the average fluorescence intensity in cells from each group. Data are expressed as mean ± SEM. ** *p *< 0.01 compared with the control group, ^# ^*p *< 0.05 compared with the cells treated with OA alone. OA, oleic acid; SMCs, smooth muscle cells; SSO, sulfosuccinimidyl oleate; SMαA, smooth muscle α-actin; SMemb, embryonic smooth muscle myosin heavy chain.

We determined the protein and mRNA levels of smooth muscle α-actin (SMαA, a marker of undifferentiated proliferating vascular SMCs) and embryonic smooth muscle myosin heavy chain (SMemb, a marker of undifferentiated proliferating vascular SMCs) in cultured vascular SMCs using immunofluorescence analysis. The protein expression of SMαA was downregulated while SMemb was upregulated in vascular SMCs treated with OA at a concentration of 250 μmol/L (both *p *< 0.01, Figure 3A and 3B). However, treatment with SSO significantly attenuated OA-induced changes in the expression of SMαA and SMemb (both *p *< 0.05, Figure 3A and 3B).

### OA enhances atherosclerotic lesion development

**Figure 4:**
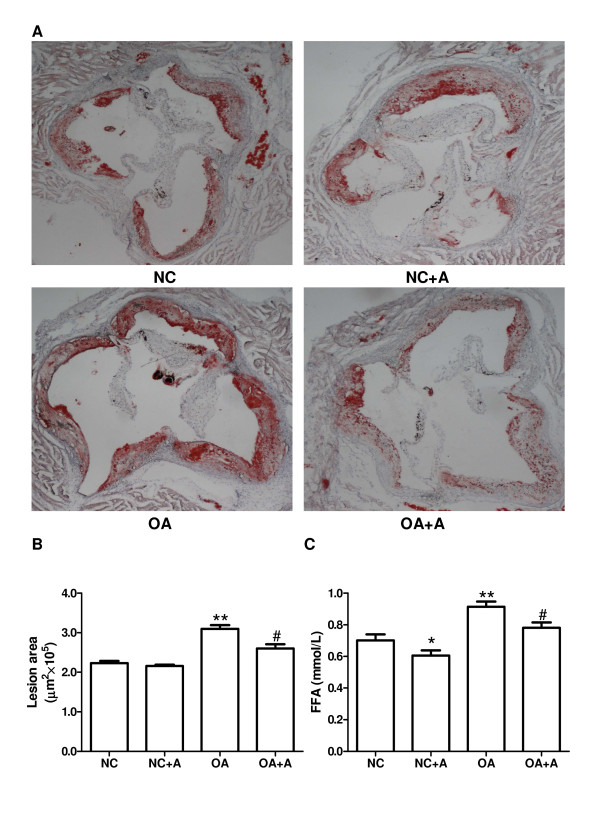
**OA enhances atherosclerotic lesion development**. (A) Oil Red O staining of atherosclerotic lesions in the aortic root at the level of the aortic valves. Magnification ×40. (B) Quantitative analyses of the proximal aorta atherosclerotic lesion areas in the normal chow (NC), NC plus acipimox (NC+A), oleic acid (OA) and OA plus acipimox (OA+A) groups. (C) Plasma levels of FFA in the four groups. Data are expressed as mean ± SEM. n = 6 mice per group. * *p *< 0.05, ** *p *< 0.01 compared with the control group, ^# ^*p *< 0.05 compared with the cells treated with OA alone. NC, normal chow; OA, oleic acid; A, acipimox; FFA, free fatty acid.

Oil Red O stained sections of the aortic root from ApoE^-/- ^mice fed with normal chow (NC) or OA showed that OA-fed mice had increased lesion area compared to NC-fed mice (2.23 ± 0.06 ×10^5 ^μm^2 ^vs. 3.09 ± 0.10 ×10^5 ^μm^2^, *p *< 0.01, Figure 4A and 4B). This effect was partially reversed by acipimox (OA: 3.09 ± 0.10 ×10^5 ^μm^2 ^vs. OA+A: 2.60 ± 0.10 ×10^5 ^μm^2^, *p *< 0.05, Figure 4A and 4B). In addition, oral administration of acipimox did not influence the atherosclerotic lesion of ApoE^-/- ^mice fed with NC (NC: 2.23 ± 0.06 ×10^5 ^μm^2 ^vs. NC+A: 2.16 ± 0.03 ×10^5 ^μm^2^, *p *> 0.05, Figure 4A and 4B). In OA-treated ApoE^-/- ^mice, the plasma FFA levels in the fasting state were elevated compared with mice fed with a NC diet (0.70 ± 0.04 mmol/L vs. 0.91 ± 0.03 mmol/L, *p *< 0.01, Figure 4C). OA-induced increases in plasma FFA levels were partially reversed by acipimox (OA: 0.91 ± 0.03 mmol/L vs. OA+A: 0.78 ± 0.03 mmol/L, *p *< 0.05, Figure 4C). Additionally, in ApoE^-/- ^mice fed with a NC diet, the level of plasma FFA was also decreased by treatment with acipimox (NC: 0.70 ± 0.04 mmol/L vs. NC+A: 0.60 ± 0.03 mmol/L, *p *< 0.05, Figure 4C).

## Discussion

There are two novel findings in the present study. First, OA induces smooth muscle foam cell formation in a dose dependent manner and enhances atherosclerotic lesion development in ApoE^-/- ^mice. Second, the atherogenic effects of OA are CD36 dependent. These findings suggest that FFA directly causes the formation of vascular SMC derived foam cells and accelerates atherosclerosis through CD36.

There have been a number of recent studies that have demonstrated the existence of SMC derived foam cells in atherosclerotic lesions [15]. However, the understanding of underlying mechanisms has been very limited [16]. There have been numerous efforts to establish *in vitro *approaches to induce smooth muscle foam cell formation, but with only limited success [3-5]. Vascular SMCs accumulate only small amounts of cholesteryl esters when exposed to modified LDL [4]. This failure to overaccumulate cholesteryl esters is due to an LDL-mediated downregulation of cell surface LDL receptors [17]. These results demonstrated that SMCs are insensitive to LDL. Using a saturated fatty acid, OA, we successfully reproduced, *in vitro*, the major biochemical and morphological alterations that occur within SMCs *in vivo *during atherosclerosis. The current study developed an effective and convenient way to induce the formation of smooth muscle foam cells.

It has been accepted that nonesterified FFA contributes to cardiovascular mortality and is an independent risk factor of atherosclerosis [9]. However, the mechanisms by which FFA causes the development of atherosclerotic lesion are unknown. Previous studies have demonstrated that FFA activates the immune system and promotes a proinflammatory state, which may contribute to its proatherogenic action [18]. The present study provides a direct mechanism by which FFA can deliver significant lipid accumulation to SMCs and induce foam cell formation. The CD36 is one of a number of fatty acid transporters that mediate the uptake of FFA by adipocytes and muscle cells. The current findings provide direct evidence that CD36 is a key determinant of the atherogenic actions of OA. CD36 is not only present on vascular SMCs but also on the surface of monocytes/macrophages. Additionally, CD36 is also involved in uptake of proatherogenic modified forms of LDL. A previous study demonstrated that the absence of CD36 on macrophages may protect against atherosclerosis in ApoE^-/- ^mice [19], strongly supporting a pro-atherogenic role for CD36. These results provide new insights into the pro-atherogenic mechanisms of CD36 by mediating the uptake of FFA other than modified LDL uptake. CD36 might become a novel target for the prevention and treatment of atherosclerosis.

We also demonstrated that elevated plasma levels of FFA in ApoE^-/- ^mice can accelerate atherosclerosis, which confirmed the atherogenic action of FFA *in vivo*. We addressed whether reducing FFA levels ameliorates atherosclerosis. Most FFA in plasma is derived from hydrolysis of adipose tissue triglyceride stores. Acipimox, as an antilipolytic agent, is an effective agent for lowering plasma FFA [20]. We found that acipimox significantly reduced plasma FFA levels and attenuated OA-induced enhancement of atherosclerotic lesions in ApoE^-/- ^mice on an OA diet. In addition, acipimox remarkably decreased plasma FFA levels in ApoE^-/- ^mice on a NC diet, but did not influence the development of atherosclerotic lesions. This finding suggests that acipimox may ameliorate atherosclerosis by lowing plasma levels of FFA that have been elevated. Therefore, acipimox might become a potent agent for the treatment of atherosclerosis associated with elevated plasma FFA levels.

## Conclusions

In conclusion, the present results demonstrate that OA induces smooth muscle foam cell formation and enhances atherosclerotic lesion development via CD36. Moreover, the OA-induced acceleration of atherosclerosis can be reversed by reducing plasma FFA levels using the antilipolytic agent acipimox. The current study also provides a model of smooth muscle foam cells for the study of atherosclerosis. The present findings may contribute to mechanistic insights into fatty acid-induced atherosclerosis.

## Methods

### Animal care

Male Wistar rats, 6 to 8 weeks of age, were obtained from a local animal center. ApoE^-/- ^mice were purchased from the Model Animal Research Center of Nanjing University. Animals were housed under a 12 h/12 h day/night cycle with *ad libitum *food and water. Experimental procedures were approved by the Hospital Animal Care and Use Committee.

### Aortic SMC culture

Aortic SMCs were obtained from the thoracic aorta of Wistar rats and cultured using previously described tissue explant methods [21]. SMCs were grown in high-glucose Dulbecco's modified Eagle's medium (DMEM; Hyclone, Logan, UT, USA) supplemented with 10% fetal calf serum (FBS; Hyclone, Logan, UT, USA). Cultures were maintained at 37°C in a humidified atmosphere of 95% air/5% CO_2_. The smooth muscle phenotype of cultured cells was verified by positive immunofluorescence for smooth muscle specific α-actin.

### SSO synthesis

SSO was synthesized according to the method reported earlier by Isenberg *et al *[22]. 0.25 mmol of OA, 0.25 mmol of N-hydroxysulfosuccinimide and 0.275 mmol of dicyclohexylcarbodiimide were reacted in 0.5 mL anhydrous N, N-dimethylformamide overnight at room temperature. After crystallizing out the dicyclohexylurea, the solution was filtered and chilled. Then, the product was precipitated out of solution by the addition of cold ethyl acetate and dried under vacuum.

### Induction of smooth muscle foam cell formation

Cultured vascular SMCs were plated on cover slides in six-well plates and maintained in culture media until the cells reached 95% confluence. Experiment 1: The cells were incubated for 48 hours with 0, 100, 250 and 500 μmol/L of OA in DMEM culture medium. After that, the cells were washed with phosphate-buffered saline, fixed with 4% paraformaldehyde, and stained with Oil Red O, which detects neutral lipids such as cholesteryl esters and triglycerides. Intracellular triglyceride and cholesteryl esters were detected. Experiment 2: The cells were divided into three groups: control (treated with vehicle), OA (treated with 250 μmol/L of OA), and OA+SSO (treated with 250 μmol/L of OA plus 250 μmol/L of SSO). After incubation for 48 hours, Oil Red O staining of the cells was performed, and the intracellular triglyceride and cholesteryl esters were measured. The cells were collected for the detection of CD36 expression.

### Western blot analysis

Cell lysate was prepared by lysing the cells with buffer containing 1% Triton X-100, 150 mM NaCl, 1 mM EDTA, 2.5 mM sodium pyrophosphate, 1 mM β-glycerophosphate, 1 mM Na_3_VO_4_, 1 μg/mL leupeptin, 1 μg/mL aprotinin, and 20 mM Tris (pH 7.5) [23]. The lysates were centrifuged to remove cell debris. Protein concentration was determined by Bio-Rad protein assay reagent (Bio-Rad Laboratories, USA). Lysate was electrophoretically transferred onto a nitrocellulose membrane and immunoblotted with rabbit anti-rat CD36 IgG (1:500 dilution, monoclonal, Sigma-Aldrich Co., USA). The blots were incubated with a horseradish peroxidase-conjugated secondary antibody (1:1000 dilution, Santa Cruz Biotechnology, USA), and the bound antibody was visualized using a colored reaction. The relative band intensity was quantified by the use of Quantity One software (Bio-Rad). Equal loading of protein was confirmed by measuring β-actin expression.

### Semi-quantitative reverse-transcription-PCR (RT-PCR)

Total RNA was extracted from cells with Tripure reagent (Roche Diagnostics Corp., Indianapolis, IN, USA) according to manufacturer instructions [24]. Semiquantitative RT-PCR for CD36 was performed as described previously. The primers for rat CD36 mRNA were as follows: 5'-GTG CAA AGA AGG AAA GCC-3' (sense) and 5'-CAT CAC TAC TCC AAC ACC-3' (antisense). The primers for rat β-actin mRNA were as follows: 5'-GAA GAT CCT GAC CGA GCG TG-3' (sense) and 5'-CGT ACT CCT GCT TGC TGA TCC-3' (antisense). Equal loading of RNA was confirmed by measuring β-actin expression. The PCR products were visualized by electrophoresis in 1% agarose gel (Bio-Rad, CA, USA) with ethidium bromide (0.5 μg/ml; Sigma, MO, USA).

### Immunofluorescence

Cultured vascular SMCs were rinsed with PBS, fixed with ice-cold acetone for 15 minutes, incubated in blocking solution (10% FBS) for 20 minutes at room temperature, and incubated with rabbit anti-SMαA or rabbit anti-SMemb (1:200 dilution, Santa Cruz Biotechnology, USA) overnight at 4°C. The cells were washed and incubated with antibody conjugated to a fluorescent probe (rhodamine-conjugated goat anti-rabbit IgG or FITC-conjugated goat anti-rabbit IgG) (1:100 dilution, Santa Cruz Biotechnology, USA) for 30 min at room temperature. After removing the unbound secondary antibodies by washing the preparations with PBS, imaging was performed using a fluorescence microscope (Nikon TE2000, USA).

### Animal treatment

Male ApoE^-/- ^mice, 6 to 8 weeks of age, were randomized to the NC, OA, or OA plus lipolysis inhibitor acipimox (OA+A) groups. OA diet contains 10% oil. Mice in OA+A group were fed OA diet and treated by oral gavage with 20 mg/kg/day of acipimox. The administration period lasted 4 weeks.

### Assessment of atherosclerosis

The heart with aorta was fixed overnight in paraformaldehyde and then embedded in optimal cutting temperature cryosectioning medium. Atherosclerotic fatty streak lesions in the aortic root were analyzed using Oil Red O staining and were counterstained with hematoxylin [25]. Atherosclerotic lesions were captured as digital images by a color camera attached to a microscope (Nikon TE2000, USA), and total mean lesion area was quantified using image analysis software.

### Plasma FFA level measurement

Fasting blood samples were obtained and plasma FFA was determined by a microplate enzymatic assay using a commercially available kit (Jiancheng Bioengineering Institute, Nanjing, China). The measurements were performed according to manufacturer guidelines.

### Statistical analysis

Continuous data are presented as mean ± SEM. Comparisons between groups were determined by one-way ANOVA with a post hoc Student's t-test (SPSS Inc., Chicago, IL). Probabilities of *p *< 0.05 were considered statistically significant.

## List of abbreviations

FFA: free fatty acid; FAT/CD36: fatty acid translocase; SMCs: smooth muscle cells; OA: oleic acid; NC: normal chow; LDL: low density lipoprotein; SSO: sulfo-N-succinimidyl oleate; SMαA: smooth muscle α-actin; SMemb: embryonic smooth muscle myosin heavy chain.

## Competing interests

The authors declare that they have no competing interests.

## Authors' contributions

SM carried out the animal studies and drafted the manuscript. DY carried out the cell studies. DL participated in the molecular studies. BT participated in the design of the study and performed the statistical analysis. YY conceived of the study, and participated in its design and coordination and helped to draft the manuscript. All authors read and approved the final manuscript.
